# Supplementary figures and images for: An acetylation-mediated chromatin switch governs H3K4 methylation read-write capability
Source: eLife. 2023 May 19;12:e82596. doi: 10.7554/eLife.82596 (PMC10229121; doi:10.7554/eLife.82596)

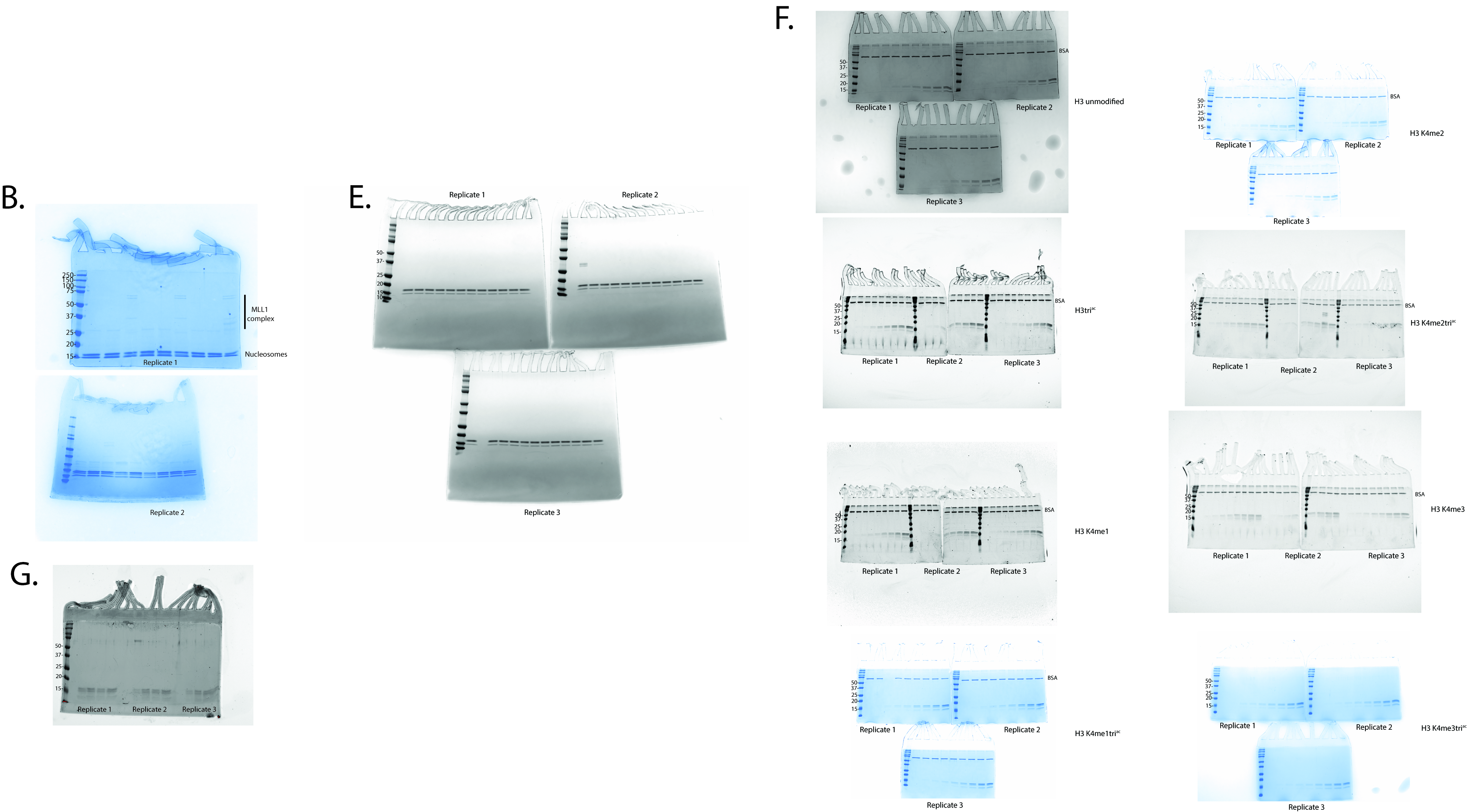

Supplement: Figure 2—figure supplement 1—source data 2. [file elife-82596-fig2-figsupp1-data2.zip › Figure 2-Figure supplement 1 zipped/Figure 2-Figure supplement 1 unedited gels labeled 03172023.tif]

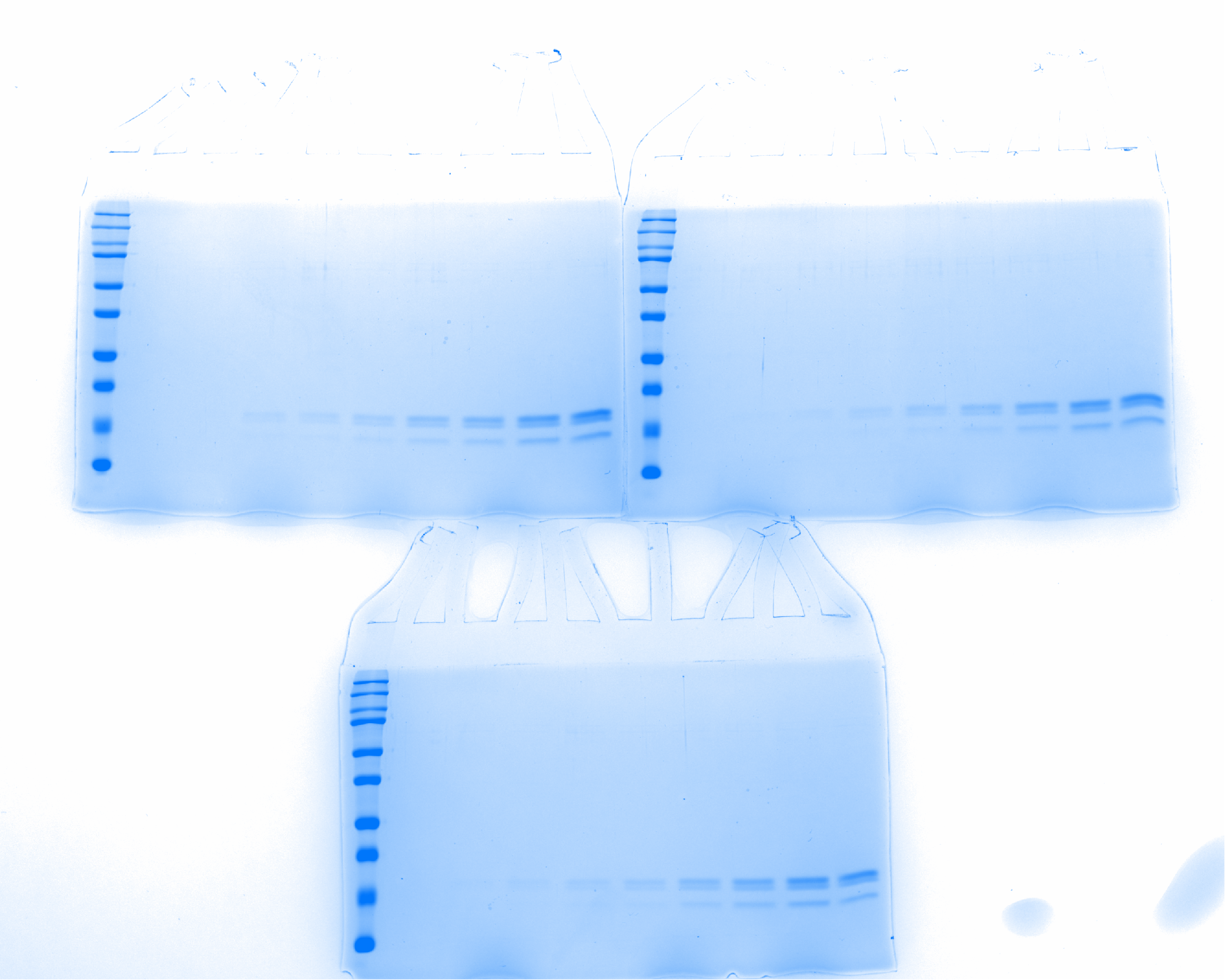

Supplement: Figure 2—figure supplement 1—source data 2. [file elife-82596-fig2-figsupp1-data2.zip › Figure 2-Figure supplement 1 zipped/Figure 2-Figure supplement 1 Panel F H3 K4me3triac_RAW..tif]

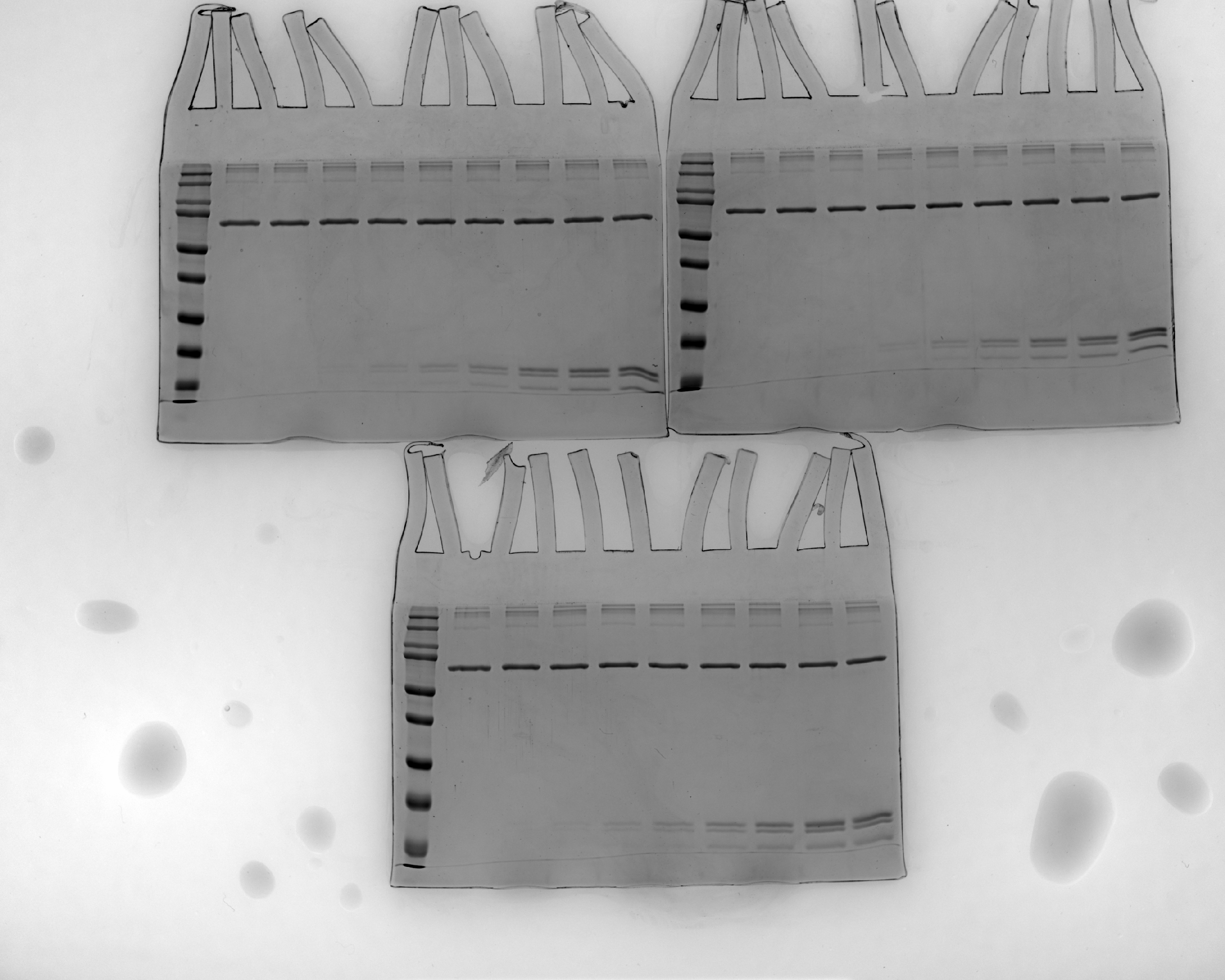

Supplement: Figure 2—figure supplement 1—source data 2. [file elife-82596-fig2-figsupp1-data2.zip › Figure 2-Figure supplement 1 zipped/Figure 2-Figure supplement 1 Panel F H3 unmodified_RAW.tif]

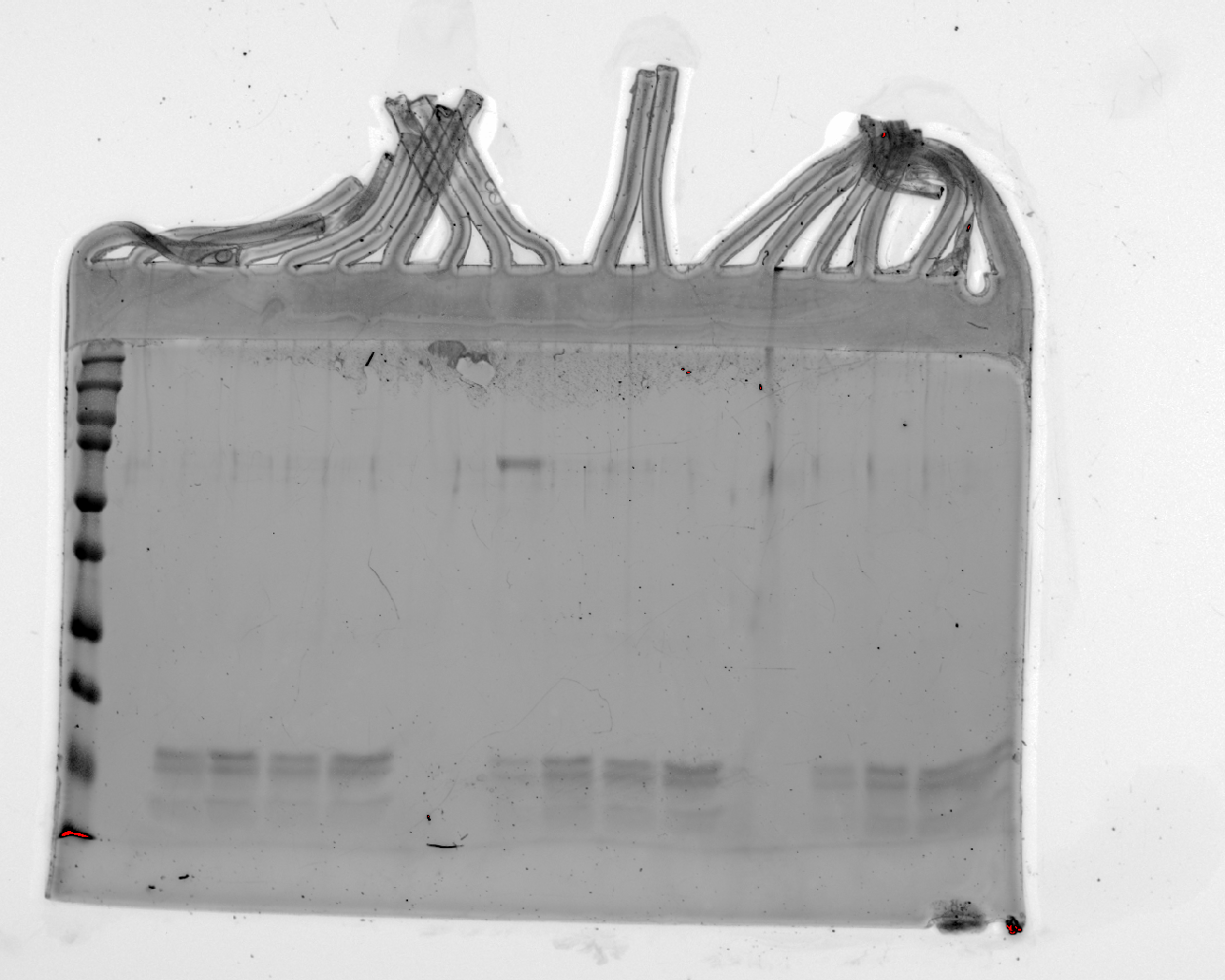

Supplement: Figure 2—figure supplement 1—source data 2. [file elife-82596-fig2-figsupp1-data2.zip › Figure 2-Figure supplement 1 zipped/Figure 2-Figure supplement 1 Panel G heteronucs_RAW.tif]

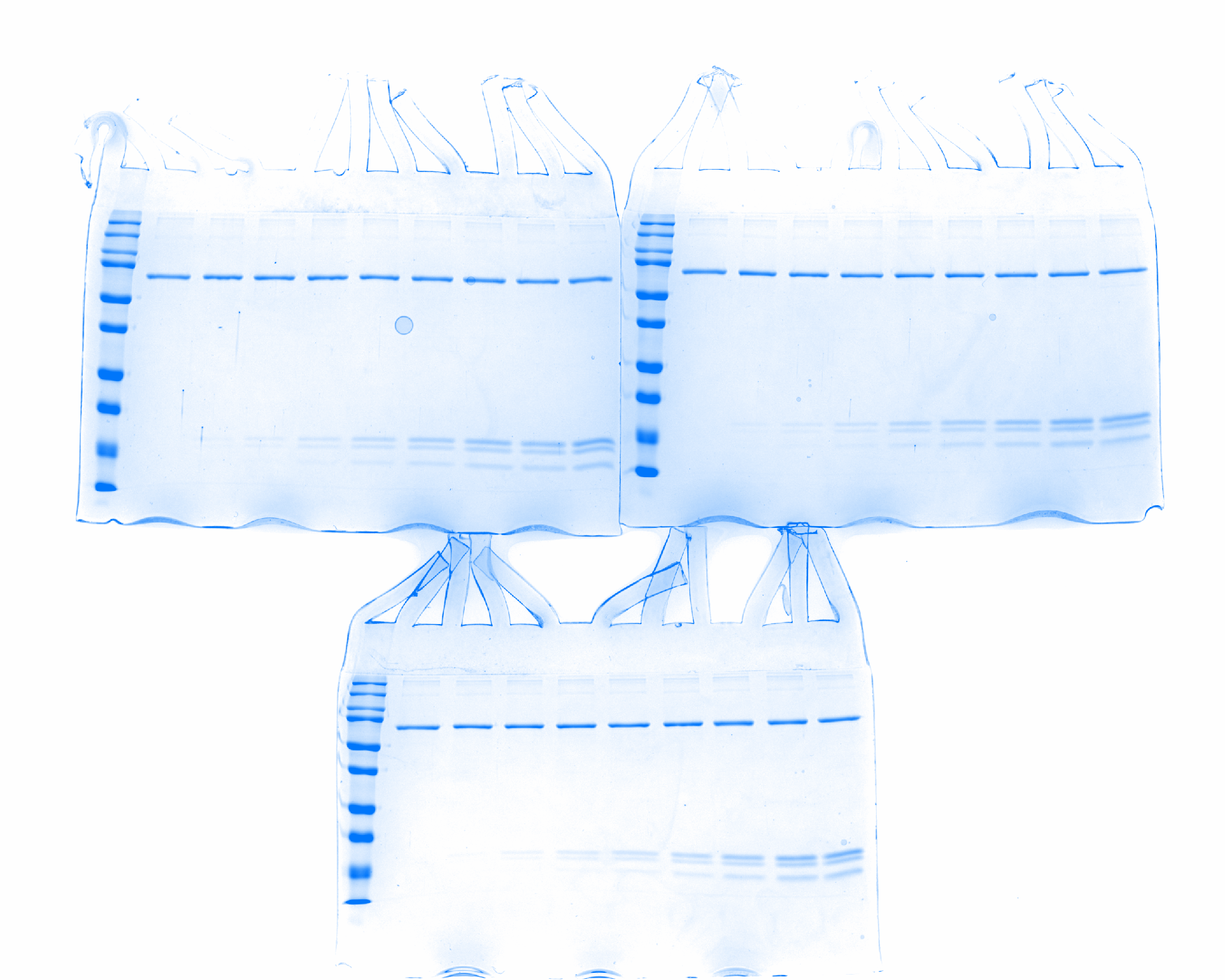

Supplement: Figure 2—figure supplement 1—source data 2. [file elife-82596-fig2-figsupp1-data2.zip › Figure 2-Figure supplement 1 zipped/Figure 2-Figure supplement 1 Panel F H3 K4me2_RAW..tif]

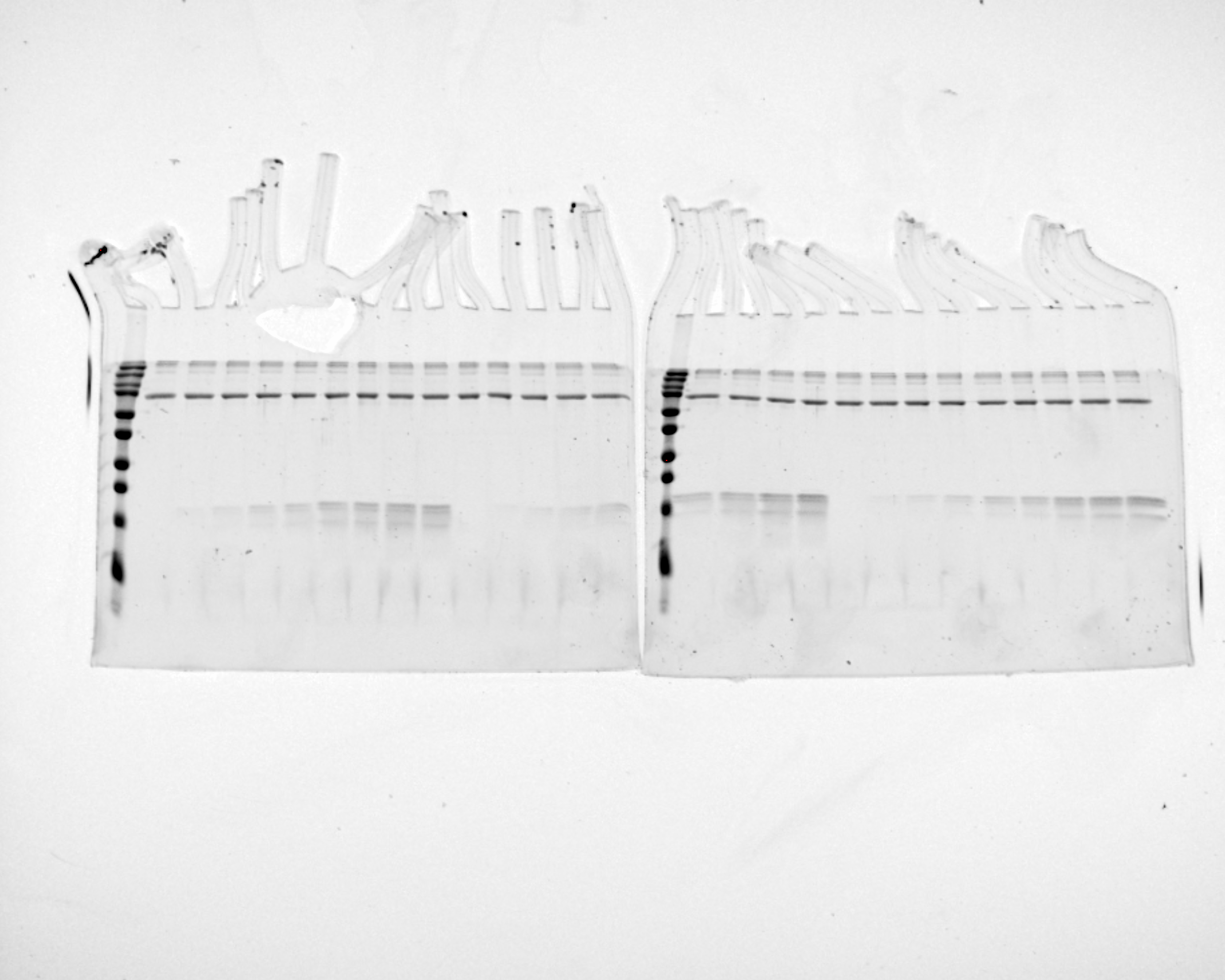

Supplement: Figure 2—figure supplement 1—source data 2. [file elife-82596-fig2-figsupp1-data2.zip › Figure 2-Figure supplement 1 zipped/Figure 2-Figure supplement 1 Panel F H3 K4me3_RAW..tif]

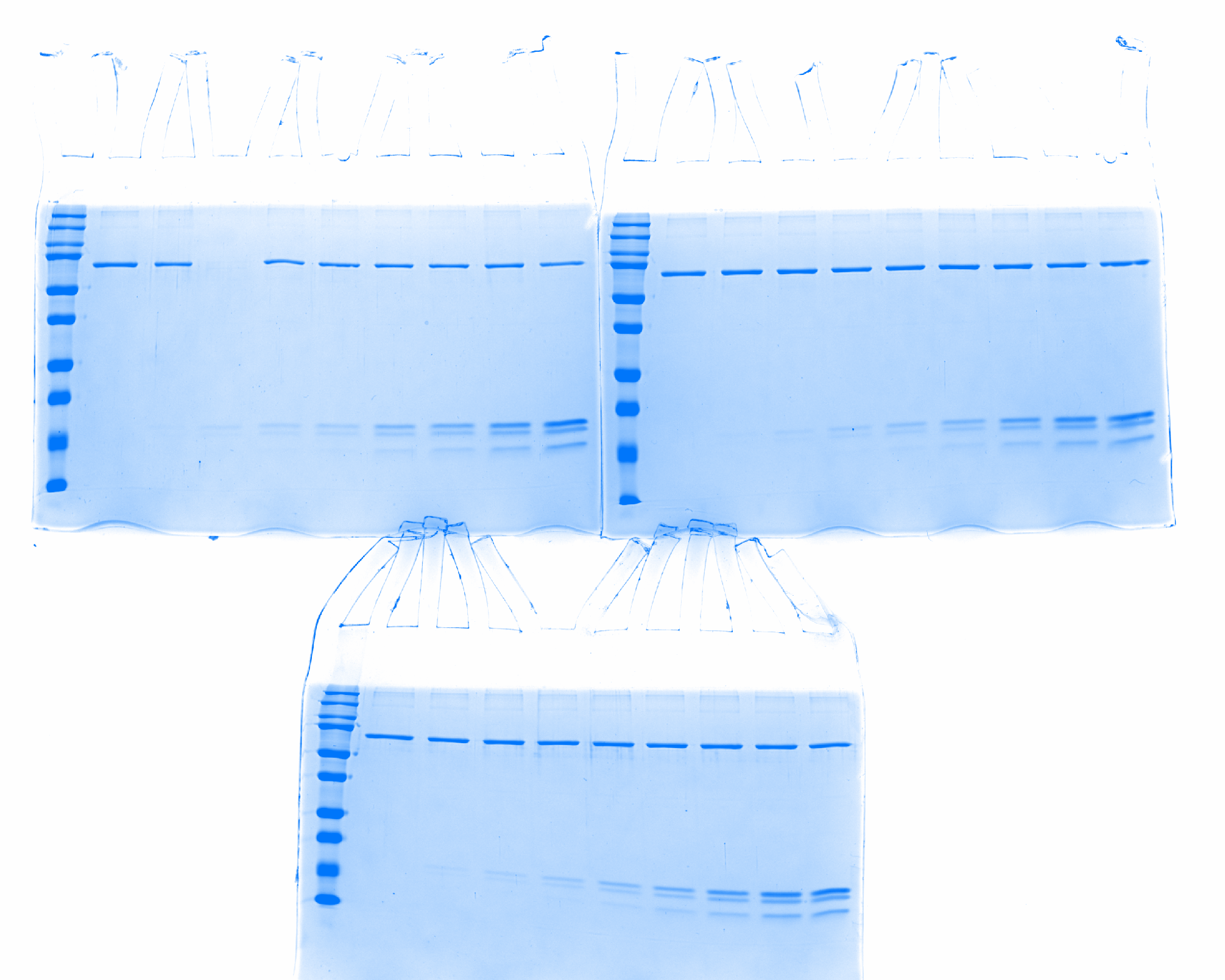

Supplement: Figure 2—figure supplement 1—source data 2. [file elife-82596-fig2-figsupp1-data2.zip › Figure 2-Figure supplement 1 zipped/Figure 2-Figure supplement 1 Panel F H3 K4me1triac_RAW..tif]

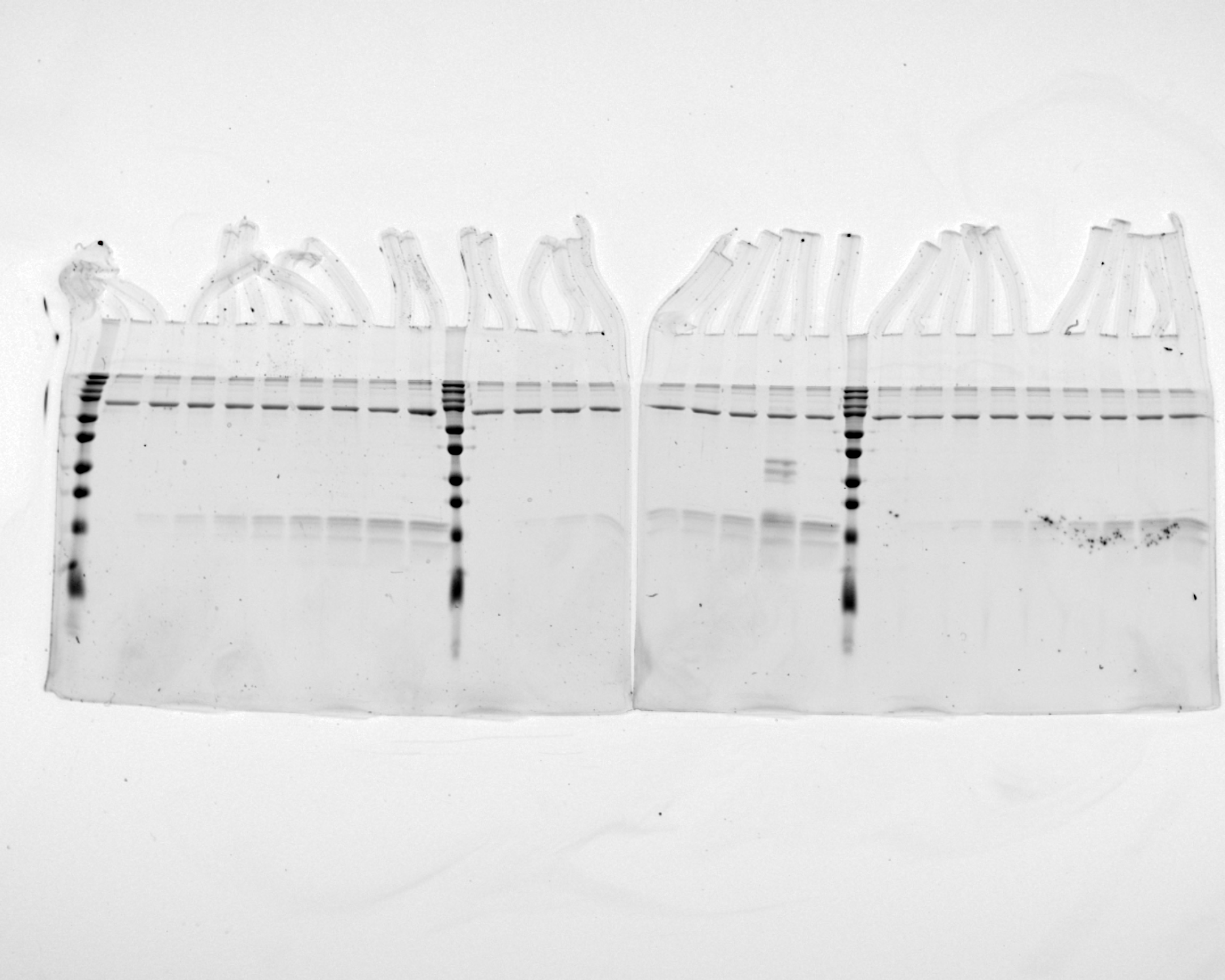

Supplement: Figure 2—figure supplement 1—source data 2. [file elife-82596-fig2-figsupp1-data2.zip › Figure 2-Figure supplement 1 zipped/Figure 2-Figure supplement 1 Panel F H3 K4me2triac_RAW..tif]

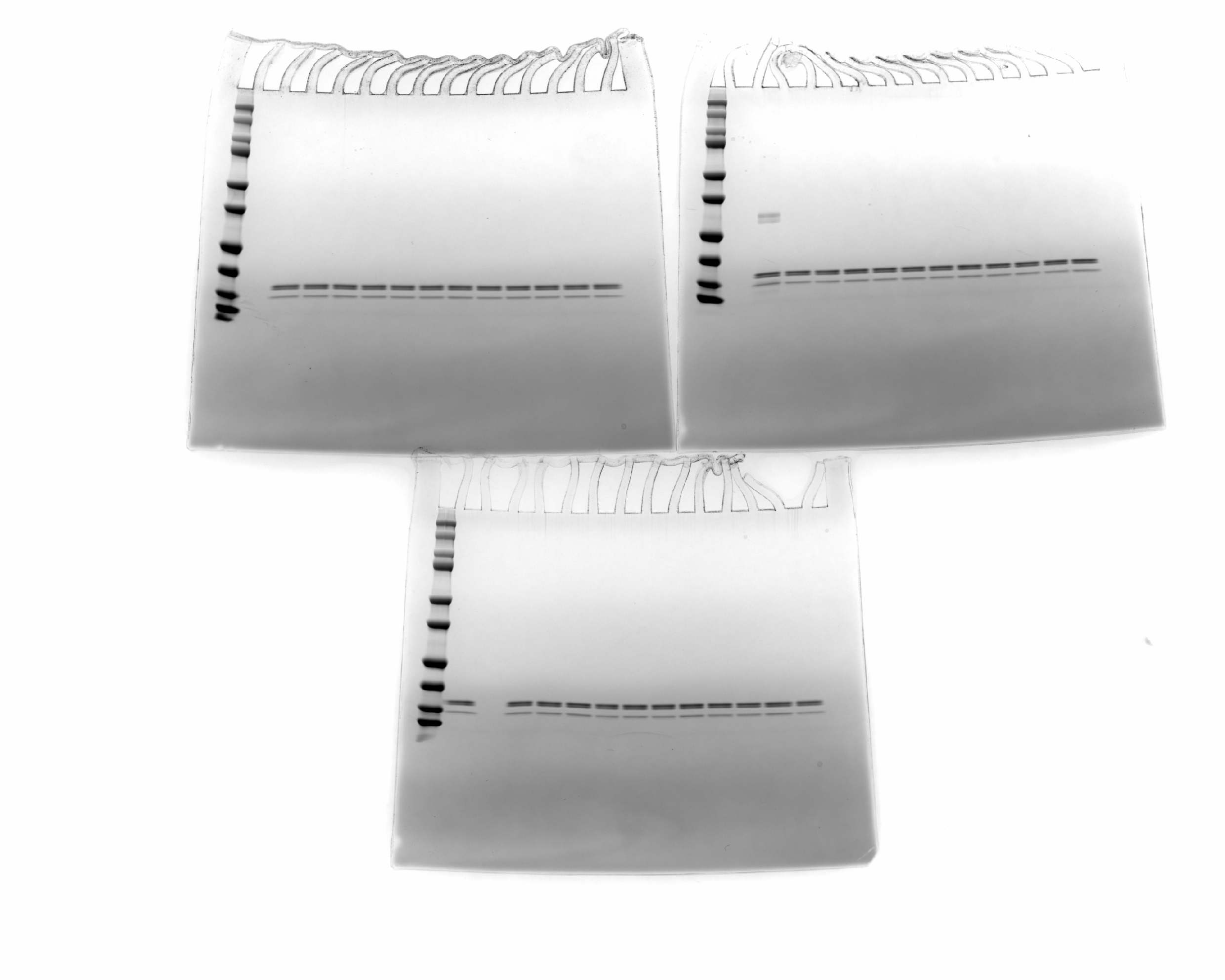

Supplement: Figure 2—figure supplement 1—source data 2. [file elife-82596-fig2-figsupp1-data2.zip › Figure 2-Figure supplement 1 zipped/Figure 2-Figure supplement 1 Panel E_RAW.tif]

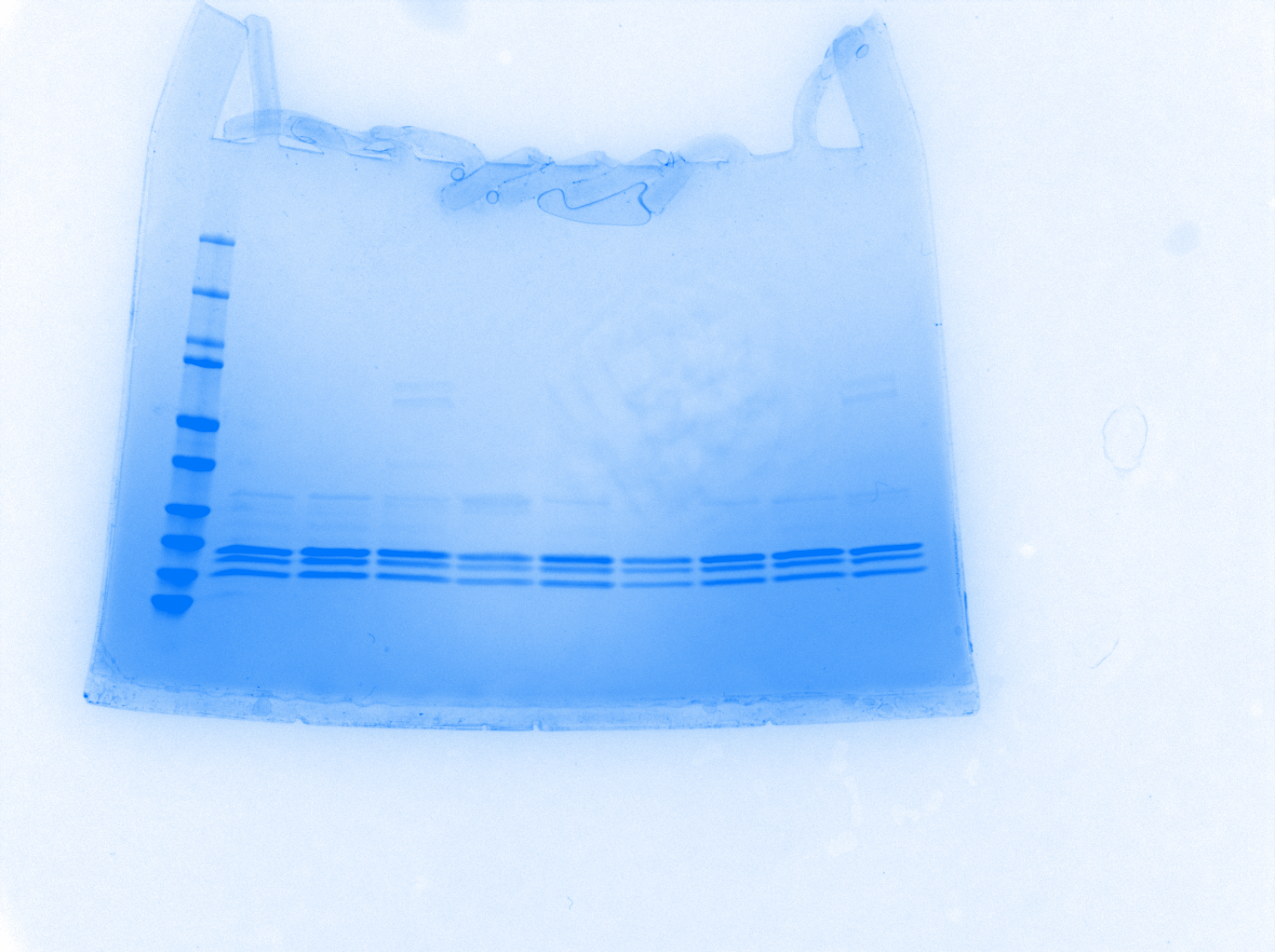

Supplement: Figure 2—figure supplement 1—source data 2. [file elife-82596-fig2-figsupp1-data2.zip › Figure 2-Figure supplement 1 zipped/Figure 2-Figure supplement 1 Panel B replicate 2_RAW.tif]

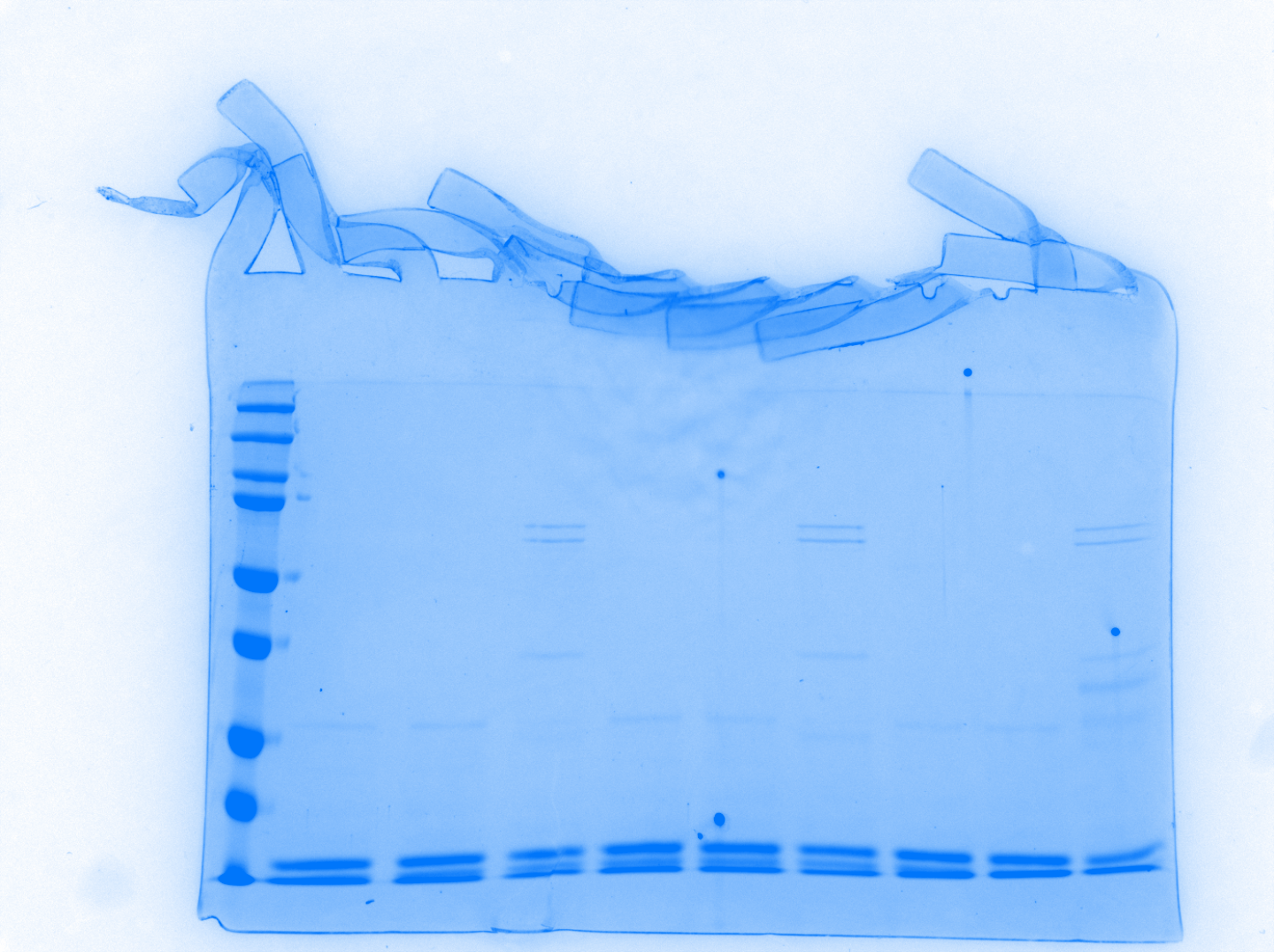

Supplement: Figure 2—figure supplement 1—source data 2. [file elife-82596-fig2-figsupp1-data2.zip › Figure 2-Figure supplement 1 zipped/Figure 2-Figure supplement 1 Panel B replicate 1_RAW.tif]

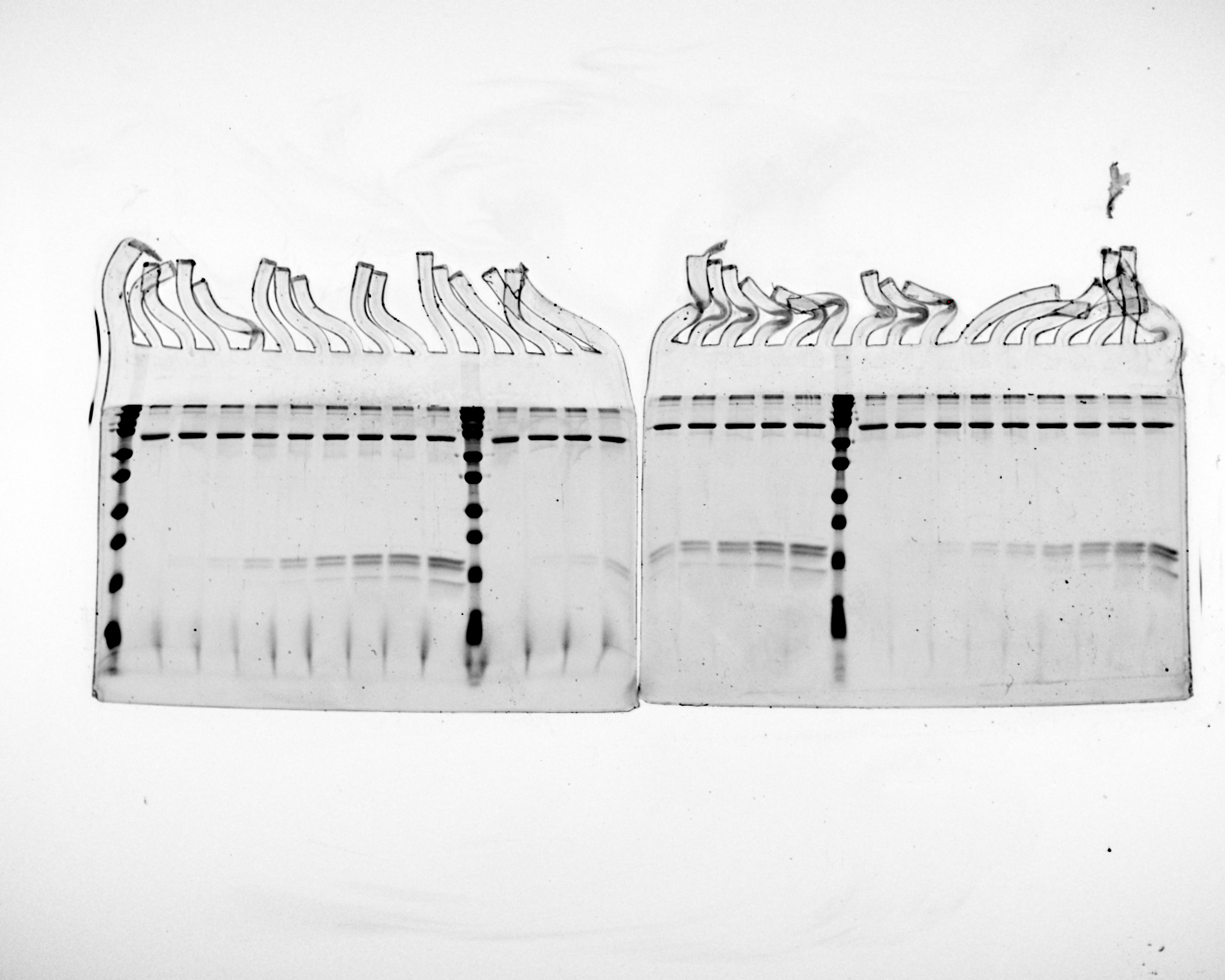

Supplement: Figure 2—figure supplement 1—source data 2. [file elife-82596-fig2-figsupp1-data2.zip › Figure 2-Figure supplement 1 zipped/Figure 2-Figure supplement 1 Panel F H3triac_RAW.tif]

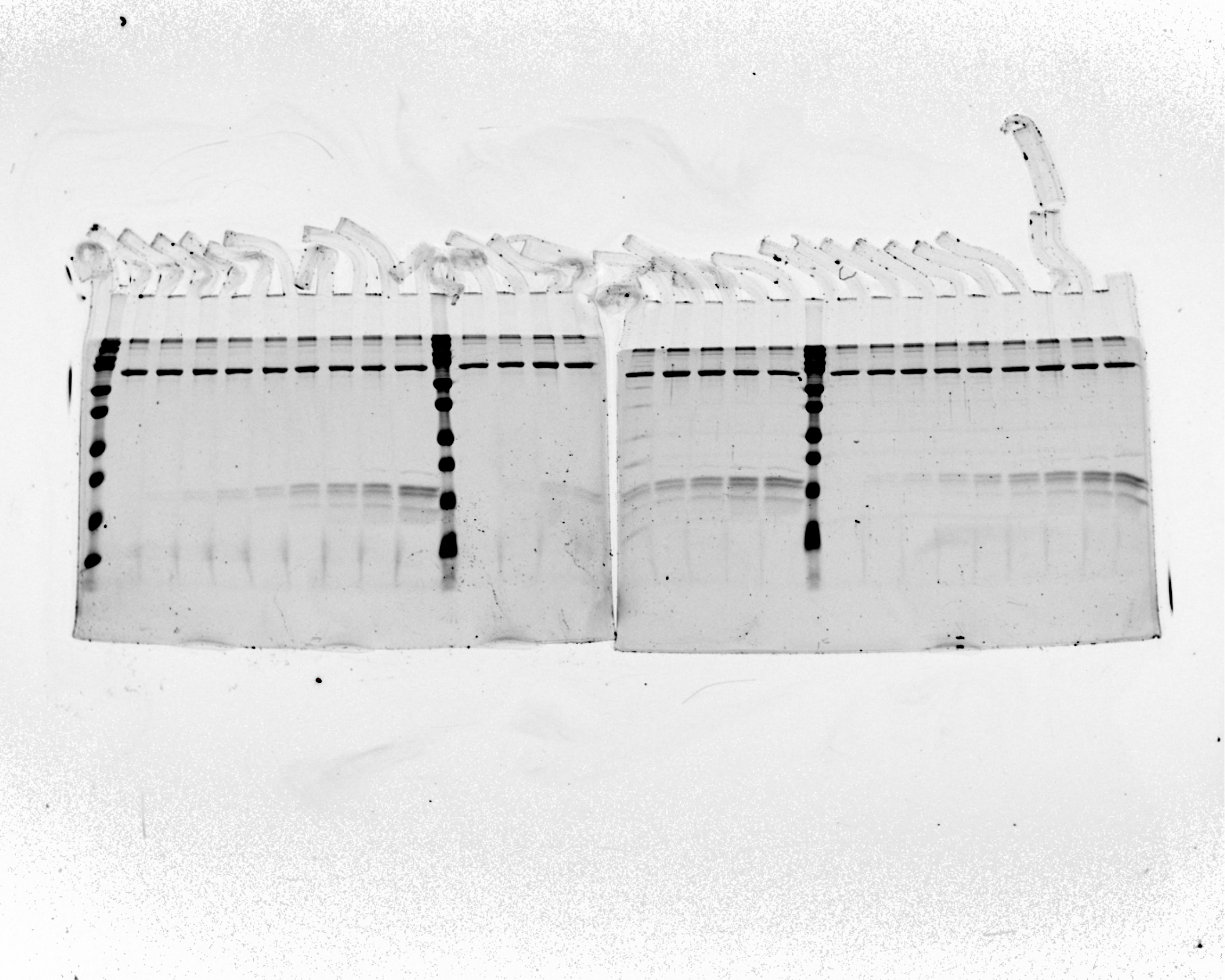

Supplement: Figure 2—figure supplement 1—source data 2. [file elife-82596-fig2-figsupp1-data2.zip › Figure 2-Figure supplement 1 zipped/Figure 2-Figure supplement 1 Panel F H3 K4me1_RAW.tif]
